# Food insecurity and the risk of depression in people living with HIV/AIDS: a systematic review and meta-analysis

**DOI:** 10.1186/s12981-020-00291-2

**Published:** 2020-06-22

**Authors:** Getinet Ayano, Light Tsegay, Melat Solomon

**Affiliations:** 1 Research and Training Department, Amanuel Mental Specialized Hospital Addis Ababa, PO BOX 1971, Addis Ababa, Ethiopia; 2 School of Public Health, Curtin University, Perth, WA Australia; 3 College of Health Sciences, Axum University, Axum, Ethiopia

**Keywords:** Food insecurity, Depression, HIV, AIDS, Systematic review, Meta-analysis

## Abstract

**Background:**

The link between food insecurity and depression in people living with HIV/AIDS (PLWHA) has been explored in numerous studies; however, the existing evidence is inconclusive due to inconsistent results. Therefore, the objective of this systematic review and meta-analysis is to examine the relationship between food insecurity and depression in PLWHA.

**Methods:**

We systematically searched PubMed, EMBASE, and Scopus to identify relevant studies. A random-effect model was used for conducting the meta-analysis. We assessed the risk of publication bias by funnel plot and Egger’s regression asymmetry test.

**Results:**

In this review, seven studies were included in the final analysis. Our meta-analysis revealed that food insecurity significantly increased the risk of depression in PLWHA [RR 2.28 (95% CI 1.56–3.32)]. This association remained significant after adjusting for the confounding effects of drug use [RR 1.63 (95% CI 1.27–2.10)], social support [RR 2.21 (95% CI 1.18–4.16)] as well as ART drugs [RR 1.96 (95% CI 1.17–3.28)]. Our subgroup and sensitivity confirmed the robustness of the main analysis.

**Conclusion:**

This systematic review and meta-analysis suggest a significant association between food insecurity and increased risk of depression PLWHA. Therefore, early screening and management of food insecurity in PLWHA seem to be necessary.

## Introduction

According to the U.S. Department of Agriculture, food insecurity refers to “a lack of consistent access to food for an active, healthy life” [1]. Food insecurity is a considerable problem in both developed and developing countries with greater prevalence in developing than developed countries [2–4]. For example, previous studies showed that in 2016 about 15.6 million households in American [5] and 52% of households in South Africans in 2005 were found to be food insecure households [6].

Epidemiologic evidence also showed that the reported prevalence of food insecurity is remarkably high in people living with HIV/AIDS (PLWHA) and is associated with poor HIV health outcomes [7, 8]. For instance, A 2009 study conducted in India found that 56% of PLWHA were food insecure at the time of enrollment to the study [9]. In another study conducted in Russia, the prevalence of food insecurity among PLWHA was 46% [10].

A substantial body of evidence has linked food insecurity in PLWHA to an increased risk of depression [11, 12]. For example, a 2011 study conducted in Uganda found that food-insecure PLWHA who were food insecure were found to be 2.83 times more likely to develop major depressive disorders as compared to those PLWHA who were food secure [13]. In a more recent study conducted in the USA, those PLWHA who were in a very low food insecure stage were found to be 4.19 times more likely to develop depression as compared to food secure PLWHA [11].

Although the above epidemiologic studies found a greater risk of depression in PLWHA who were food insecure, these results are not constant all over the available studies; there are also articles that reported no significant risk of depression in PLWHA [14]. Thus, the objective of this systematic review and meta-analysis is to examine the relationship between food insecurity and depression in PLWHA in order to conclude the association and formulate implications for future epidemiologic research and clinical practice.

## Methods

### Research method and designs

We conduct this systematic review and meta-analysis in accordance with the preferred reporting items for systematic review and meta-analysis guidelines (PRISMA) [15]. We utilized a pre-defined protocol for search strategy, data extraction, study selection, as well as analysis.

### Data source and searches

Three authoritative electronic databases (EMBASE, PubMed, and Scopus) were systematically searched for pertinent studies. We searched without restriction on the date of publication. The systematic literature search was conducted in May 2019. The search terms and keywords included (Food insecurity OR food insufficiency) AND (HIV OR Human immune deficiency Virus OR AIDS OR Acquired immune deficiency syndrome) AND (depression OR depressive symptom OR depressive disorder). We also manually searched to identify additional relevant studies.

### Inclusion and exclusion criteria

Articles satisfying the following criteria were included in this study: First, all observational study (case–control, cross-sectional or cohort stud). Second, the exposure of interest was food insecurity. Third, the outcome was depression. Fourth, the study population was PLWHA. Fifth, studies that reported relative risks (RR) or odds ratio, estimates with the respective 95% confidence intervals (CIs), or studies that reported data to calculate these. In this review, we excluded editorials, comments, case reports, reviews, letters, abstracts presentations, as well as studies published in a non-English language.

### Data extraction

Two reviewers (MS and LT), in an independent manner, used a predesigned standard form to extract the data, which included the first author’s name, publication year, the study design, country, confounders adjusted for, risk estimate (OR/RR) and their 95% CI as suggested by PRISMA guidelines [16]. We resolved disagreements were by consensus.

### Study quality

The Newcastle–Ottawa quality evaluation scale (NOS) [17] was used to assess the quality of the included studies. The NOS scale evaluates the quality of the included studies in three areas such as comparability between the groups, recruitment of the participants, and assessment of exposure and outcome. For the cross-sectional studies, we used a modified version of NOS [18]. In fact, studies were not excluded based on the quality assessment score alone.

### Data synthesis and analysis

Comprehensive Meta-Analysis (CMA) software version3 was employed to conduct the meta-analysis [19]. In those studies that reported multiple estimates, we used the estimate with the most extensive adjustment and with the highest degree of food insecurity. To account for the heterogeneity across the studies, the random effect model was used to combine the effect estimate from included studies [20]. Q and the I^2^ statistics were used to assess heterogeneity [20]. The I^2^ statistics values such as 25, 50, and 75% represented low, moderate, and high heterogeneity respectively [21]. All the reported probabilities were two-sided. To assess the key studies that exerted a considerable impact on the heterogeneity we carried out a leave-one-out sensitivity analysis [22]. We also performed subgroup and sensitivity analysis to compare the risk between the groups as well as determining the potential source of heterogeneity between the studies. We evaluated a publication by funnel plot and Egger’s regression test [23].

## Results

### Study selection

**Fig. 1:**
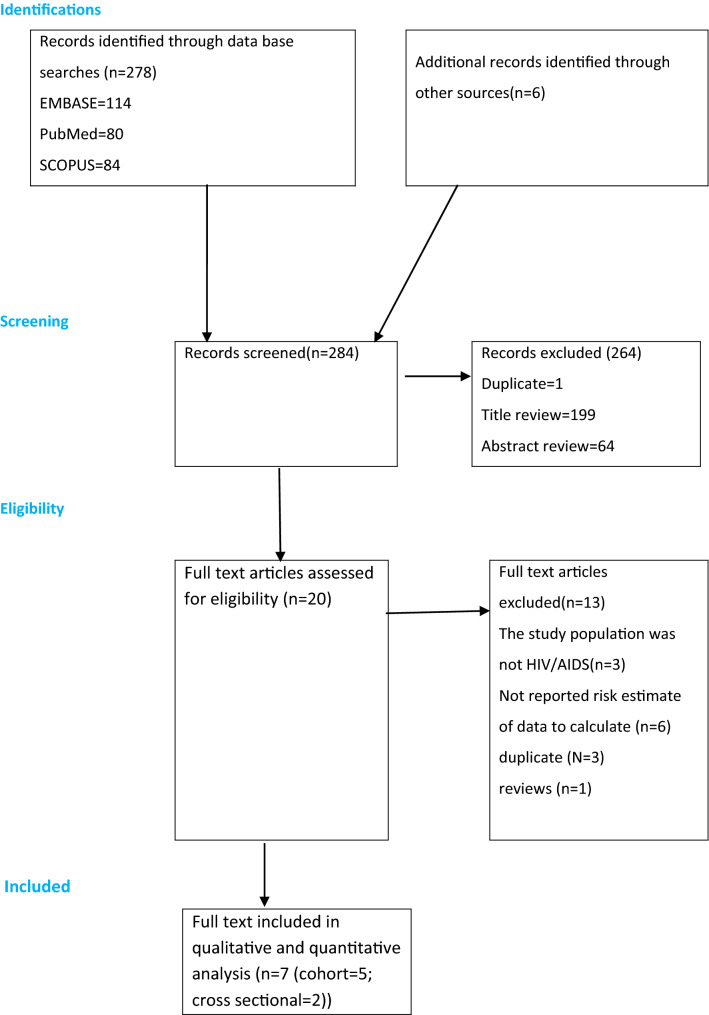
PRISMA flowchart of review search

The search strategy resulted in 278 studies. Six additional relevant references were identified through our manual search. Our review of these studies by title, duplicate, and abstract resulted in the exclusion of 264 studies, as they did not meet the predefined inclusion criteria. Our further screening of a full text of the remaining 20 articles resulted in the exclusion of further 13 studies. Therefore, seven studies were found eligible for the final analysis (Fig. 1).

### Characteristics of included studies

Table 1 shows the characteristics of the included studies. The included studies were published between December 2011 [24] and June 2018 [11]. Most of the included studies (5 studies) were conducted in the USA [11, 14, 25–27], one study conducted in Uganda [24], and one in Ethiopia [28]. Five were cohort studies [11, 14, 25–27] and two were cross-sectional studies [24, 28]. Two studies adjusted for the possible confounding effects of drug use [11, 14], two adjusted for ART drug use [11, 24], and two adjusted for social support [14, 24].

**Table 1:** The characteristics of included studies

Study name, year	Country	Study design	Measures for exposure variables	Measures for outcome variables	Crude OR/RR	Adjusted OR/RR	Adjusted for
Palar et al. 2018 [11]	USA	Prospective cohort	HFSSM	CESD	Not available	2.39 (1.63–3.42) (Marginal FI)	Not available
						3.18 (2.14–7.73) (low FI)	
						4.19 (2.79–6.30) (very low FI)	
Kaplusky et al. 2015 [14]	USA	prospective cohort study	Radimer/corner questionnaire	Burnam depression screen	2.15 (1.11–5.55)	1.5 (0.6–3.7)	Social support, emotional support, poverty and drug use
Palar et al. 2015 [27]	USA	Prospective cohort study	HFIAS	BDI–II	Not available	1.41 (0.99–2.02) (Mild FI)	Sex, baseline depression, race, educational status, ART drug use, emergency visits, recent homelessness, heavy drinking, illicit drug use
						1.34 (1.02–1.78) (Moderate FI)	
						1.64 (1.26–2.13) (Severe Fi)	
Kinyanda et al. 2011 [13]	Uganda	Cross sectional study	Self-report	MINI	2.83 (1.45 = 5.73)	2.89 (1.40–5.98)	Distance from HIV clinic, knowing HIV status, On ART, social support, stressful life event, stress score index
Davey-Rothwell et al. 2014 [25]	USA	Prospective study	Core Food Insecurity Module	CESD	2.91 (1.63–5.17)	2.71 (1.51–4.88)	Race, age, income, taking food stamp in last 30 days
Yeneabat et al. 2017 [28]	Ethiopia	Cross sectional study	HFIAS	CESD	5.10 (2.32–10.25)	3.83 (1.58–9.32)	Sex, age, educational status, marital status, occupational status, place of residence, number of dependent children, access to food, practice of agriculture, ownership livestock, CD4 + count, OIs
Aibibula et al. 2017 [26]	USA	Prospective cohort study	HFSSM	CESD	1.78 (1.57–2.02) (Moderate FI) 2.38 (2.14–2.65) (severe FI)	1.33 (1.20–1.48) (Moderate	Sex, age, educational status, marital status, sexual orientation, unstable housing, occupational status, clinical stage, median duration of HIV infections, OIs
						FI) 1.37 (1.25–1.51) (severe FI)	

*AIDS* Acquired Immune Deficiency Syndrome, *BDI* Beck Depression Inventory, *CES-D* Centre for Epidemiologic Studies Depression Scale Revised, *HFIAS* Household Food Insecurity Access Scale, *HFSSM* Household Food Insecurity Survey Module, *HIV* Human Immunodeficiency Virus, *MINI* Mini-International Neuropsychiatric Interview, *PLWHA* people living with human immunodeficiency virus (HIV)/Acquired immune deficiency syndrome (AIDS); *OR* odds ratio, *RR* relative risk

Regarding the tools used to assess depression, from the total, four studies used the Center for Epidemiologic Studies Depression Scale (CESD) [11, 25–28], one study used Mini-International Neuropsychiatric Interview (MINI) [24], one study used Beck depression inventory (BDI) [14] and one study used Burnam depression screen (a short form of CESD) [14]. Of these four instruments used to assess depression, three of them are screening [29–31] and one is a diagnostic instrument [32]. Regarding the instruments used to assess food insecurity, two studies used the Household Food Insecurity Access Scale (HFIAS) [27, 28], two studies used the Household Food Insecurity Survey Module (HFSSM) [11, 26], one study used Radimer/corner questionnaire of hunger and food insecurity [14], one study used Core Food Insecurity Module [25], and one study used self-reported food insecurity by participants [13].

### The quality of the included studies

In this review, we used NOS, a 9-point scoring system to evaluate the study quality. Accordingly, all the included studies were good quality studies (the NOS score for the included studies ranges between eight and nine from the total 9 points) (see Additional file 1: Table S1).

### Food insecurity and risk of depression in PLWHA

**Fig. 2:**
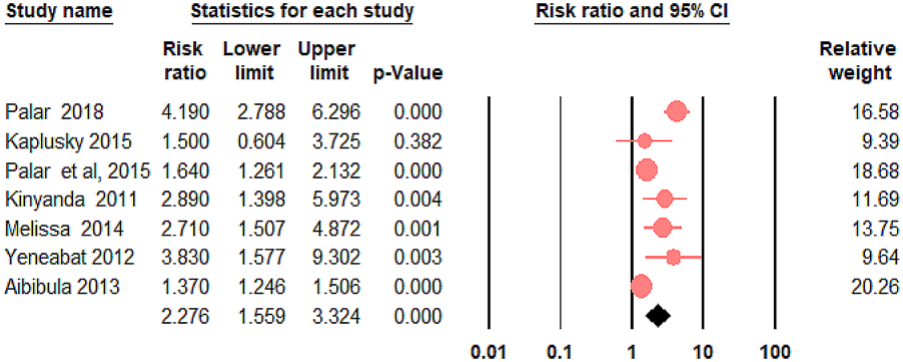
The forest plot of the association between food insecurity and depression in PLWHA

Figure 2 shows the forest plot indicating the relative risk and 95% CI of each study as well as the overall pooled relative risk. To account for the observed heterogeneity between the included studies (*I*^2^ = 84.88%; Q = 39.69; df = 6; P < 0.0001), we employed a random effect model. The meta-analysis of seven studies showed that the risk of developing depression was significantly higher in those PLWHA who were food insecure as compared to those who were food secure [RR 2.28 (95% CI 1.56–3.32)].

### Subgroups analyses by study design

**Fig. 3:**
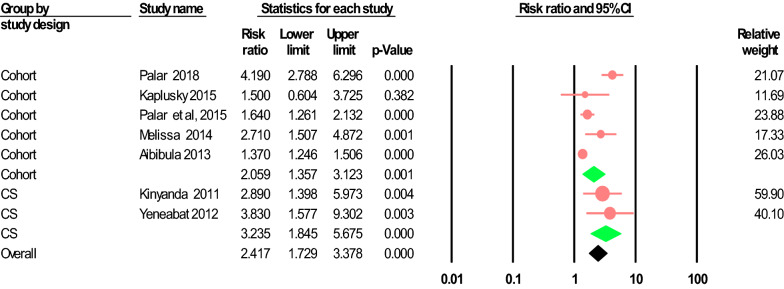
The forest plot of the association between food insecurity and depression in PLWHA. Subgroup analysis by study design

In our subgroup analysis by the study design, the risk of developing depression based on cohort studies was 2.06 (95% CI 1.36–3.12)), whereas based on cross-sectional studies was 3.24 (95% CI 1.85–5.68). The heterogeneity was significant for cohort studies (*I*^2^ = 87.62%; Q = 42.30; df = 4; p < 0.0001), but not for cross-sectional studies (*I*^2^ = 0.00%; Q = 0.23; df = 1; p = 0.630) (see Fig. 3).

### Subgroup analyses by the level of food insecurity

In our stratified analysis by the level of food insecurity, the risk of depression was 1.83 (95% CI 1.09–3.07)), 1.95 (95% CI 0.85–4.52)), 2.59 (95% CI 1.03–6.48)) respectively for mild, moderate and severe food insecurity. The observed differences in risk estimates between the groups were not statistically significant (P = 0.813) (see Table 2).

**Table 2:** Summary of the subgroup and Sensitivity analysis of all studies based on type of the severity food insecurity, adjustment for drug use, ART and social support and quality of the included studies

Subgroups	Studies, n	Relative risk (%)	95% CI	Heterogeneity across the studies		Heterogeneity between the groups (P value)
				I^2^	P value	
Level of food insecurity						0.813
Mild	2	1.83	1.09–3.07	75.28	0.044	
Moderate	2	1.95	0.85–4.53	81.34	0.021	
Severe	2	2.59	1.03–6.48	93.02	< 0.001	
Adjustment for drug use						0.134
Adjusted	2	1.63	1.27–2.10 2	0.00	0.850	
Not adjusted	5	2.71	1.46–5.00	89.83	< 0.001	
Adjustment for social support						0.902
Adjusted	2	2.21	1.18–4.16	18.02	0.269	
Not adjusted	5	2.31	1.48–3.63	89.07	< 0.001	
Adjustment for ART						0.599
Adjusted	2	1.96	1.17–3.28	51.67	0.269	
Not adjusted	5	2.43	1.32–4.48	88.93	< 0.001	

### Publication bias

**Fig. 4:**
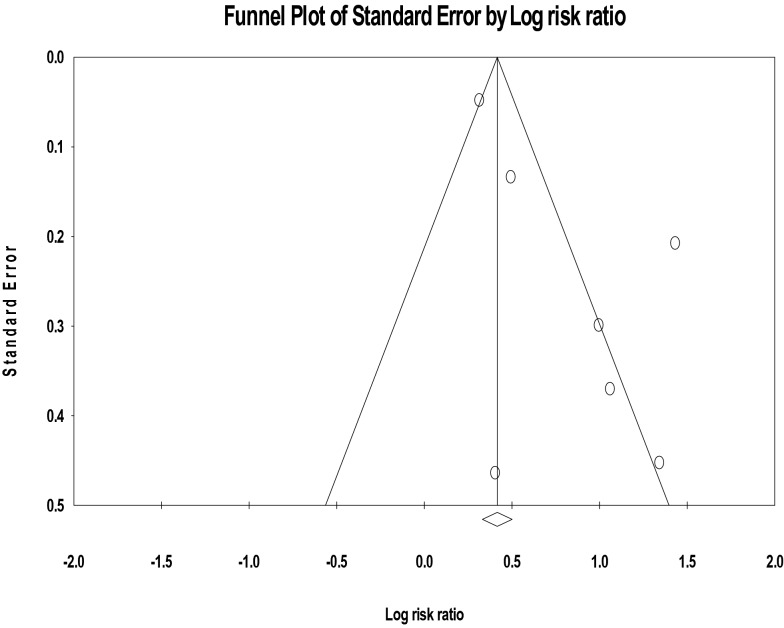
The risk of publication bias

The funnel plot and Egger’s regression tests (B = 2.60, SE = 0.96, P = 0.042) provided evidence of substantial publication bias for the association between food insecurity and the risk of depression in PLWHA (Fig. 4).

### Sensitivity analysis

To identify the possible effects of drug use, we conducted stratified analysis by restricting the analysis to the studies that adjusted for the potential confounding effects of drug use. In this analysis, an increased risk of depression was observed in PLWHA who were food insecure in studies that accounted for the effects of drug use [RR 1.63 (95% CI 1.27–2.10)] as it was in the studies with no adjustment [RR 2.71 (95% CI 1.46–5.00)]. In this analysis, the observed difference in risk estimates of depression (RR) between the groups was not significant (P = 0.134). The reported heterogeneity across the studies was also not significant in studies that adjusted for the confounding effects of drug use (*I*^2^ = 0.00%; Q = 0.034; df = 1; p = 0.850) but it was significant in those that did not adjust for the effects of drug use (I^2^ = 89.83%; Q = 39.30, df = 4, P < 0.001) (see Table 2).

We further conducted the sensitivity analysis by restricting the analysis to studies that adjustment for confounding effects of lack of social support. We found an increased risk of depression in PLWHA who were food insecure in studies that accounted for the effect of social support [RR 2.21 (95% CI 1.18–4.16)] as well as in studies that did not account for confounding effects of social support [RR 2.32 (95% CI 1.48–3.63)]. This analysis resulted in no significant heterogeneity between studies that adjusted for social support (*I*^2^ = 18.02%; Q = 1.22; df = 1; p = 0.269) but it was significant in those that did not adjust for social support (I^2^ = 89.07%; Q = 36.61, df = 4, P < 0.001) (see Table 2).

Finally, we performed the analysis by restricting the analysis to studies that accounted for potential confounding effects of ART drug treatment because evidence suggests that antiretroviral therapy side effects were positively associated with depression in PLWHA in previous studies  [33]. We found an increased risk of depression in PLWHA who were food insecure in studies that accounted for the effect of ART drugs [RR 1.96 (95% CI 1.17–3.28)] as well as in studies that did not account for the effect of ART drugs [RR 2.43 (95% CI 1.32–4.48)]. The heterogeneity between studies that adjusted for ART was not significant (*I*^2^ = 51.67%; Q = 2.06; df = 1; p = 0.150) but it was significant in those studies that did not adjust ART drugs (I^2^ = 88.93%; Q = 36.14, df = 4, P < 0.001) (see Table 2).

We also conducted a leave-one-out sensitivity analysis for further examining the possible cause of heterogeneity in the analysis of food insecurity and depression in PLWHA. This analysis resulted in a pooled estimated relative risk (RR) ranging between 1.88 (95% CI 1.41–1.70) and 2.58 (95% CI 2.52–4.29) after the deletion of a single study. This finding indicates that our findings were robust and not dependent on a single study (Additional file 2: Table S2).

## Discussion

### Main findings

This systematic review and meta-analysis assessed the risk of depression in PLWHA who were food insecure across five cohorts and two cross-sectional studies. Our final analysis demonstrated that there was a positive and significant association between food insecurity and greater risk of depression in PLWHA (RR = 2.28) that was unaffected by the level of adjustment for ART drug use, the degree of social support, as well as substance use (drug use). When we limit the analysis by the level of food insecurity, the risk was higher for severe food insecurity (RR = 2.59) followed by moderate (RR = 1.95) and mild food insecurity (R = 1.83), which supports the robustness of the main analysis. This finding suggests the necessity of the application of early screening and intervention strategies of food insecurity in PLWHA.

However, the included cross-sectional studies, the sample sizes, and the level of adjustment for the potential confounding factors must be considered. The level of adjustment factors was inconsistent in the included seven studies. Drug use (2 studies), age of participants (4 studies), on ART (2 studies), and social support (2 studies) were the most common potential confounders taken into account in the included studies. Only one study accounted for the possible confounding effects of the previous history of depression [27]. This study found a significant and week association for moderate food insecurity and moderate association for severe food insecurity, but the association was not significant for mild food insecurity. This result suggests the possibilities that the association seen in studies with a lower level of adjustment could be due to chance or the effects of confounding. Supporting this view, a substantial body of research showed a greater risk of depression in those PLWHA who substance users were, had a previous history of depression, as well as poor social support. Thus, in studies, which did not account for the effect of the above factors, the observed association between food insecurity and greater risk of depression could be due to the confounding effect of unmeasured drug use, ART drugs as well as lack of social support.

In fact, the robustness of an increased risk of depression in PLWHA with food insecurity was supported by our s analysis that we conduct in the current study: Firstly, the robustness of the observed association between food insecurity and depression was supported by our dose–response analysis. In this analysis, we found a greater risk of developing depression in those participants with severe food insecurity followed by moderate and mild food insecurity. These findings suggest the possible causal association between food insecurity and depression. Secondly, the robustness of the association observed in the current study was also supported by the sensitivity analyses that we conducted restricting the analysis to studies that controlled the confounding effects of drug use, ART drugs, and social support. In this analysis, we found the increased risk of depression in those PLWHA studies which accounted for the possible effects of drug use [RR 1.63 (95% CI 1.27–2.10)] social support [RR 2.21 (95% CI 1.18–4.16)] as well as those studies that adjusted for effects of ART drugs [RR 1.96 (95% CI 1.17–3.28)].

### Differences among the studies included in the meta-analysis

Even though our meta-analysis resulted in a significant association between food insecurity and depression in PLWHA, the observed differences across the seven studies led to a significant level of between-study heterogeneity in our final analysis. The stage of the disease, the country, and the exposed population differed on a number of characteristics which may have contributed to the variance in the risk of depression in those PLWHA who were food insecure. Nevertheless, our leave-one-out sensitivity analysis indicated that the risk of developing depression remained virtually unchanged from the main analysis. This finding suggests that our findings were strong and not significantly influenced by a single study. Additionally, our subgroup analysis and sensitivity analysis by the degree of food insecurity as well as the level of adjustment to a range of confounding factors supported the robustness of our findings. Moreover, in order to make the finding of the current meta-analysis meaningful, we have used a random-effects model to pool risk estimates from the individual studies. It is widely held that the summary effect estimates in the random effect model meta-analysis are more conservative than fixed effect summaries in the epidemiological meta-analysis, suggesting the robustness of our findings [20].

In our study, two of the evidence came from cross-sectional studies therefore the possibility that depression in PLWHA may lead to rather than precede the diagnosis of food insecurity might be considered. This is because epidemiologic evidence reported a reciprocal relationship between food insecurity and depressive symptoms [34]. Supporting our view, scientific studies have found that depressive symptoms were significantly associated with food insecurity [34–36]. In fact, this may not be a major concern in our study because when we limit our analysis to cohort studies where exposure (food insecurity) certainly precede the outcome (depression), we found a significant association between food insecurity and the increased risk of depression [RR 2.06 (95% CI 1.36–3.12)].

### Possible mechanisms

There is a range of explanations for the associations between food insecurity and increased risk of depression in PLWHA. Firstly, food insecurity is linked with incomplete HIV viral load suppression and less immune reconstitution in PLWHA, which in turn linked with a higher risk of depression [37–39]. Secondly, food insecurity is also associated with a significant reduction in CD4 count, which has been consistent, associated with a greater risk of depression in PLWHA in previous studies [40–42]. Thirdly, the rates of underweight are higher in food-insecure PLWHA as compared to food-secure people and underweight has been associated with a higher risk of depression in several epidemiologic studies [43, 44]. Furthermore, food insecurity is associated with a higher risk of opportunistic infections and other comorbid conditions, which are among the major risk factors of depression among PLWHA [45, 46]. Finally, food insecurity is associated with reduced social capital, and higher levels of (or increased level of) social isolation, stigma, stress, and loneliness, which are in turn linked with increased risk of depression in those food-insecure people [47, 48].

### Strength and limitations

This systematic review and meta-analysis have several strengths: Firstly. First, we utilized a predesigned search strategy, data abstraction, quality assessments, analysis, data extraction, and quality assessment to minimize the reviewer’s bias. Secondly, we performed a sensitivity and subgroup analysis based on drug use, level of social support, and use of ART drugs to identify the small study effect and the risk of heterogeneity in analyses of the risk of depression and those with food insecurity. Thirdly, we use a standard tool to evaluate the quality of the included studies (NOS) and our evaluations showed that the methodologic qualities of the included studies were good.

However, the current review also has some limitations: (1) our meta-analysis resulted in significant associations between food insecurity and depression, but the degree of adjustments was inconsistent across the studies and important confounding variables including the effects of drug use, social support, previous history of depression, as well as ART drugs, were not adjusted in most studies. (2) Our final analysis showed significant heterogeneity between the studies. However, the observed variations between the studies may not be a major issue because our sensitivity and subgroup analysis resulted in a greater risk of depression in those food-insecure participants in both cohort 2.06 (95% CI 1.36–3.12)) and cross-sectional 3.24 (95% CI 1.85–5.68) studies. (3) The inclusion of a relatively low number of studies in our subgroup and sensitivity analysis is the other limitation of this review, which might reduce the precision of the reported estimate. (4) Pertinent studies published in a language other than English may have been missed. Finally, we found a significant publication bias the possibility of publication bias indicating the possibilities of remained studies due they may not be reported or published based on their findings or other factors. (5) We have not conducted the literature search in PsycINFO to identify studies relevant to the current review, which is one of the authoritative databases in mental health. In fact, we have conducted our systematic search for relevant studies in the three commonly used databases in mental health such as EMBASE, PubMed, and Scopus. We have also tried to include all the potential articles relevant to the review by our manual search and we believe that we have included the required important articles to estimate the pooled relative risk for the association between food insecurity and the risk of depression in people living with HIV/AIDS.

## Conclusion

This systematic review and meta-analysis suggest food insecurity is associated with an increased risk of depression in PLWHA. Early screening for food insecurity and depression is warranted in PLWHA.

## **Supplementary information**

**Additional file 1: Table S1.** The quality of the included studies based on NOS score (9 point score).

**Additional file 2: Table S2.** The sensitivity analysis of food insecurity and the risk of depression in PLWHA after each study removed.

## Data Availability

All data generated and analyzed during this study are included in this review.

## Abbreviations

AIDS: Acquired Immune Deficiency Syndrome
BDI: Beck Depression Inventory
CES-D: Centre for Epidemiologic Studies Depression Scale-Revised
HFIAS: Household Food Insecurity Access Scale
HFSSM: Household Food Insecurity Survey Module
HIV: Human Immunodeficiency Virus
MINI: Mini-International Neuropsychiatric Interview
OR: Odds Ratio
PLWHA: People living with human immunodeficiency virus (HIV)/Acquired immune deficiency syndrome (AIDS)
RR: Relative risk
