# Supplementary material for: Food insecurity and the risk of depression in people living with HIV/AIDS: a systematic review and meta-analysis
Source: AIDS Res Ther. 2020 Jun 22;17:36. doi: 10.1186/s12981-020-00291-2 (PMC7310141; doi:10.1186/s12981-020-00291-2)
Supplement: Supplementary file 1 — Additional file 1: Table S1. The quality of the included studies based on NOS score (9 point score). [file 12981_2020_291_MOESM1_ESM.docx]

**Table S1**: the quality of the included studies based on NOS score (9 point score)

| Study name, year | NOS score | Remarks |
| --- | --- | --- |
| Palar et al, 2018, [25] | 9 | Good |
| Kaplusky et al, 2015, 3 | 9 | Good |
| Palar et al, 2015, 4 | 9 | Good |
| Kinyanda et al, 2011, [24] | 9 | Good |
| Melissa et al, 2014, 6 | 9 | Good |
| Yeneabat et al, 2017,9 | 8 | Good |
| Aibibula et al, 2017, 1 | 8 | Good |
