# Supplementary material for: Food insecurity and the risk of depression in people living with HIV/AIDS: a systematic review and meta-analysis
Source: AIDS Res Ther. 2020 Jun 22;17:36. doi: 10.1186/s12981-020-00291-2 (PMC7310141; doi:10.1186/s12981-020-00291-2)
Supplement: Supplementary file 2 — Additional file 2: Table S2. The sensitivity analysis of food insecurity and the risk of depression in PLWHA after each study removed. [file 12981_2020_291_MOESM2_ESM.docx]

**Table S2**: The sensitivity analysis of food insecurity and the risk of depression in PLWHA after each study removed.

| Study name, year | RR | 95%CI |
| --- | --- | --- |
| Palar et al, 2018 | 1.88 | 1.41-2.52 |
| Kaplusky et al, 2015 | 2.38 | 1.56-3.59 |
| Palar et al, 2015 | 2.49 | 1.45-4.29 |
| Kinyanda et al, 2011 | 2.20 | 1.47-3.30 |
| Melissa et al, 2014 | 2.21 | 1.47-3.34 |
| Yeneabat et al, 2017 | 2.15 | 1.46-3.18 |
| Aibibula et al, 2017 | 2.58 | 1.70-3.94 |
| Key: The analysis is based on random effect model | | |
